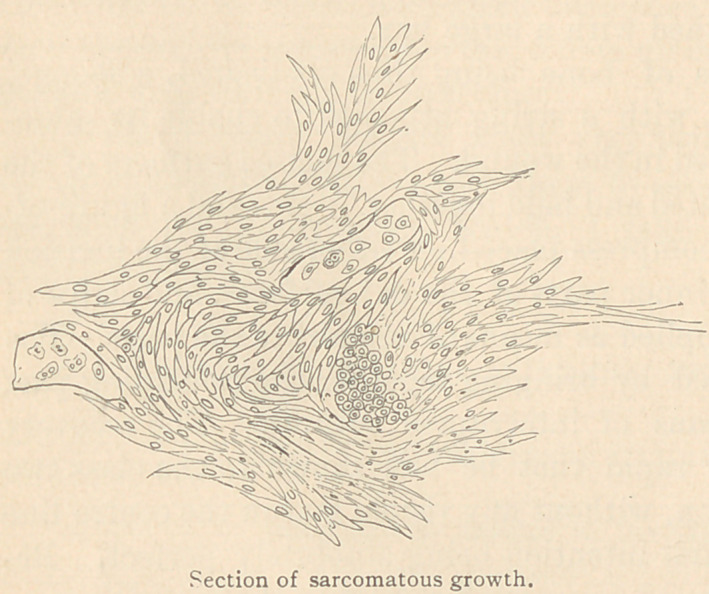# Clinic of Prof. Garretson

**Published:** 1887-06

**Authors:** Robert S. Ivy


### HOSPITAL OF ORAL SURGERY, PHILADELPHIA.

### CLINIC OF PROFESSOR GARRETSON.

REPORTED BY ROBERT S. IVY, D. D. S.

An operation involving removal of the greater portion of the left superior maxilla was recently performed at the above clinic. The patient, a young man sent by his physician from New York, presented an enlargement of the bone, of a malignant character, over the antrum and alveolar process, but the extent to which the growth had involved the surrounding bones could not be ascertained. The patient having been etherized to a point ensuring insensibility during the first stages of operating and to permit more complete examination, a cut was made in the median line of the upper lip dividing the coronary artery, which was ligated; the incision was continued around the wing of the nose and upward in a straight line towards the inner part of the orbit, thence outward to the side of the face, terminating above the maxilla-malar articulation. In dissecting away the flap relieved by this incision, the facial and infra-orbital arteries were cut. The external part of the diseased bone having been thus exposed and an opening made into the antrum, more complete examination was made by the professor and his *confrère* and assistant, Dr. Cryer, resulting in the discovery that the whole of the bone, except the orbital plate and small portions of the malar and nasal processes, was diseased and reduced to a semi-osseous condition. With bone forceps and the surgical engine all the parts involved were cut away, exposing the back part of the antrum and the nasal and oral cavities; a lobe of the parotid gland (*socia parotidis*) could also be seen. The hemorrhage having been checked, all rough and ragged surfaces of bone, resulting from the use of the forceps, were smoothed with a large bur.

The diseased portions of bone being thus eradicated, a sponge, washed in phenol and with a string attached to enable its subsequent removal, was placed in the wound. The incised surfaces of the lip were next approximated and held together by pins and a figure-of-eight ligature, a small compress placed on either side of the incision brought in contact the inner parts of the cut surfaces, and strips of adhesive plaster were applied as means of support to the parts. The operation was completed by bringing together the remaining part of the wound, by means of interrupted sutures. The recovery of the patient was so rapid that he was shown to the class two weeks after the operation, without any visible scar in the centre line of the face, union by first intention being absolutely perfect. Referring to the old-fashioned manner of removing the superior maxilla, Professor Garretson described it as simply terrific when compared with that employed in this operation, requiring the disarticulation of the bone at all points, with the bone forceps. Modern surgery enucleated only the portion of the bone diseased, a feature specially fortunate in this case, as the orbital plate being free from disease it was left undisturbed and the retention of the patient's eyesight kept beyond question. The surgical engine with properly adapted burs, used in cases of this character, resolves apparently complicated operations into absolute simplicity.

A section of the growth was subsequently made by Dr. C. W. de Lannoy, in the pathological laboratory of the college, who says: "The examination made of the maxillary tumor will warrant its classification among the so-called myeloid sarcomata described by Corneil, Ranvier, Paget, Robin, and others. These neoplasms vary in consistence with the age of the growth. The cells are almost in absolute contact, with but a slight amount of intermediate cellular substance. The individual cells may present any or several of the forms peculiar to the sarcomata or connective tissue new formations, viz.: round, small, or large, fusiform, polygonal, and branched elements. In places the embryonic tissue may proceed to the formation of adult connective tissue, fibrous in character, and even the more complex tissue derivatives, cartilage and bone, may be found to exist.

The specimen under consideration represents the early stage of the neoplasm, that in which the inordinate production of embryonic cells has entirely replaced the osseous tissue of the maxilla, and while the general outline and dimensions of the bone are but little changed, the entire premaxilla and the maxilla anterior to the first molar are wholly destitute of any trace of bony structure. Posterior to this region, however, is seen an area more recently invaded by the neoplasm, and consequently remnants of as yet unabsorbed bony tissue are noticed.

Section of sarcomatous growth.

The spindle-shaped or fusiform cells are the most prevalent in this specimen, hence are referred to as *spindle-celled sarcoma.* Here and there, as seen in the illustration, are sections of narrow cartilaginous trabeculæ; these evince further differentiations of connective tissue, which, in time, would have become ossified.

The growth is malignant in the sense that the pathological changes alluded to usually involve, by a process of progression through continuity, the entire bone in which they first develop, stopping only at the deep layer of calcified cartilage corresponding to the articulation."

## Figures and Tables

**Figure f1:**